# Initial Requirements for the Prototyping of an App for a Psychosocial Rehabilitation Project: An Integrative Review

**DOI:** 10.3390/ijerph22020310

**Published:** 2025-02-18

**Authors:** Fagner Alfredo Ardisson Cirino Campos, Fabio Biasotto Feitosa, Marciana Fernandes Moll, Igor de Oliveira Reis, José Carlos Sánchez García, Carla Aparecida Arena Ventura

**Affiliations:** 1 School of Nursing of Ribeirão Preto, University of São Paulo (EERP-USP), Ribeirão Preto 14040-902, SP, Brazil; 2 Faculty of Psychology, University of Salamanca (USAL), 37005 Salamanca, Spain; jsanchez@usal.es; 3 Department of Psychology, Federal University of Rondonia (UNIR), Porto Velho 76801-974, RO, Brazil; fabio.depsi@unir.br; 4 Faculty of Nursing, State University of Campinas (UNICAMP-SP), Campinas 13083-970, SP, Brazil; marcfmol@unicamp.br; 5 Department of Psychiatric Nursing and Human Sciences, School of Nursing of Ribeirão Preto (EERP), University of São Paulo (USP), Ribeirão Preto 14040-902, SP, Brazil; igordeoliveirareis@usp.br (I.d.O.R.);

**Keywords:** mental health, mobile applications, projects, psychiatric rehabilitation

## Abstract

The Psychosocial Rehabilitation Project (PRP) is a tool designed to structure and organize mental health care, guided by the theoretical and practical principles of Psychosocial Rehabilitation (PR). This article aims to identify the initial requirements for the prototyping of a “Psychosocial Rehabilitation Project App”. To achieve this, an integrative review was conducted with the research question: what initial requirements are important to compose the prototype of the “Psychosocial Rehabilitation Project App” in mental health? In the search process, 834 articles were identified and exported to the online systematic review application Rayyan QCRI, resulting in 36 eligible articles for this study, along with one app. The reading of this material allowed the elicitation of three themes: privacy and data protection policy; design; and software and programming. The prototyping of the “Psychosocial Rehabilitation Project App” should prioritize data security and protection, simplicity in design, and the integration of technological resources that facilitate the management, construction, monitoring, and evaluation of psychosocial rehabilitation projects by mental health professionals.

## 1. Introduction

The Psychosocial Rehabilitation Project (PRP) is a tool that structures and organizes mental health care guided by the theoretical and practical precepts of psychosocial rehabilitation (PR). It also allows building and/or rescuing the contractuality of the psychiatric patient so that they can manage their life with autonomy, desires, values, and purposes, achieving social protagonism [1,2,3].

The literature shows that these projects, when performed on paper, are bureaucratic, fragmented, decontextualized from the psychosocial needs of the psychiatric patient, and difficult to operationalize in mental health services [4,5]. In this sense, one way to dynamize them would be to adapt them to a mobile mHealth support technology, as this resource allows streamlining traditional processes in mental health [6,7,8], promoting the exercise of human rights, citizenship, accessibility to care, and mental health services, which can reduce inequalities and social injustices [9].

Therefore, it is necessary to develop digital tools, such as mental health apps to be used by mental health professionals to support the psychosocial rehabilitation of people with severe and persistent mental disorders, seeking to promote social inclusion, the construction of autonomy, social functionality, and the exercise of citizenship in this population [10].

However, there are few apps developed for psychosocial rehabilitation, and those that exist are focused on supporting the patient through gamification [10,11].

Thus, it is necessary to develop mental health apps contextualized and adapted to the work environment and the ”real” needs regarding mental health care provided by mental health professionals [12], especially considering the context of psychosocial rehabilitation and the difficulties of the psychosocial care network inserted in the Brazilian public health system (SUS) [2,4,5,13,14].

Considering this reality and the existing gap regarding the development of apps that favor the planning of psychosocial rehabilitation by health professionals [3,11], it is essential to develop scientific investigations on this topic. The literature shows that even though there is no standard for the development of a mental health app [15,16,17,18,19], there are common steps as follows: An exploratory and descriptive literature review for researchers or developers to know the state-of-the-art about apps, identify similar apps and resources to support their idea, and gather initial prototyping requirements. Co-design or consultations with mental health professionals to contextualize the requirements of the review or to know specific requirements. The development and validations (technical, content, appearance, and usability) [4,14,15,16,17,18,19].

By following the requirements for prototyping, it is possible to develop mental health apps through the creative process, such as the “Psychosocial Rehabilitation Project App”. Prototyping allows the principal researcher to actively participate in the app’s visual design, functionality, and content, ensuring it aligns with its therapeutic purposes. This process is based on theoretical foundations that justify its therapeutic approach and is aligned with the prototyping requirements gathered from the literature review [15,20,21,22,23,24,25], content and appearance validation [23,24,26], and usability [16,27,28,29].

Thus, this article aims to identify initial requirements for the prototyping of the “Psychosocial Rehabilitation Project App”.

## 2. Materials and Methods

An exploratory, descriptive, and integrative literature review study was conducted, as adapted from PRISMA guidelines, following research stages [30,31,32,33]: Firstly, composition of the research question based on the PICo strategy. Secondly, definition of inclusion and exclusion criteria. Thirdly, selection of health sciences descriptors (DeCS) and medical subject headings (MeSH) and their respective synonyms. Fourthly, composition of the electronic search expression and its insertion into national and international databases. Finally, synthesis of the selected references. 

The research question was constructed using the PICo strategy, where P is the population/research condition, I is the intervention, and Co is the context [31]. Based on this reference, the following research question was delimited: what initial requirements are important to compose the prototype of the “Psychosocial Rehabilitation Project App” in mental health? It should be noted that P is “initial requirements”, I is “compose the prototype of the “Psychosocial Rehabilitation Project App”, and Co is “mental health”.

Considering this information, the controlled descriptors indexed in DeCS and MeSH were established, with their respective synonyms in Portuguese, English, and Spanish, and combined with Boolean operators (AND and OR) to construct the electronic search expression completed with their synonyms. The short form can be seen below, and the complete form in Table 1 [31]. P (“Design de Software” OR “Software Design” OR “Diseño de Software” OR “Projeto de Sistemas” OR “System Design” OR “Diseño de Sistemas” OR “Software” OR “Software” OR “Programas Informáticos”) AND I (“Aplicativos Móveis” OR “Mobile Applications” OR “Aplicaciones Móviles” AND “Projetos” OR “Projects” OR “Proyectos” AND “Psychiatric Rehabilitation” OR “Psychiatric Rehabilitation” OR “Rehabilitación Psiquiátrica”) AND Co (“Mental Health” OR “Mental Health” OR “Salud Mental”).

**Figure 1:**
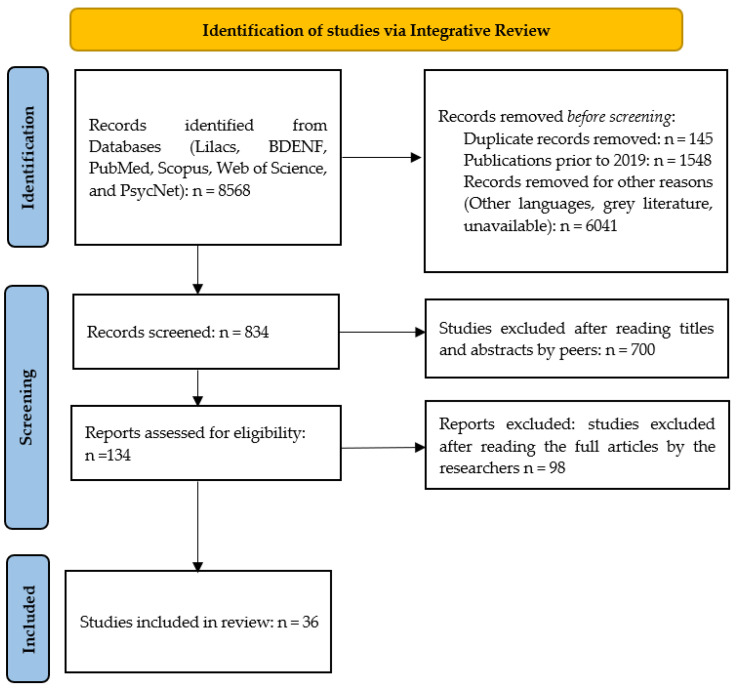
Steps flowchart for selection of articles for this integrative literature review. Source: prepared by the author (2024).

This complete electronic expression, Table 1, was inserted on 12 August 2023, into the databases of the Latin American and Caribbean Literature in Health Sciences (Lilacs), Nursing Database (BDENF), International Literature in Health Sciences (PubMed), Scopus, Web of Science, and PsycNet via remote access through the Federated Academic Community (CAFe) on the Portal of Periodicals of the Coordination for the Improvement of Higher Education Personnel (CAPES), searching by title, abstract, and keywords/DeCS/MeSH. For this purpose, complete articles published in Portuguese, English, and Spanish were included, published in the period between 2019 and 2023, corresponding to 5 years. Duplicate articles and those that did not meet the research question (eligibility) were excluded (Figure 1).

Concurrently, the 834 articles were exported to the online systematic review application Rayyan QCRI for double-blind peer review based on titles and abstracts. As there were no conflicts among the reviewers, submission to a third evaluator was not necessary [34,35]. After reading the titles and abstracts of the 834 articles with the aid of Rayyan QCRI, 700 articles were excluded for not meeting the defined eligibility criteria, resulting in 134 articles. After a thorough full-text review, 98 articles were excluded, leaving 36 articles for inclusion in this study. These articles were re-read and synthesized to address the research question and objectives of this work (Table 2).

To complement the integrative literature review and gain a better understanding of technological products (apps) related to psychosocial rehabilitation apps, a search was conducted during the same period as the review (between 2019 and 2023) in Google Patents (N = 0), Espacenet (N = 1), Latipat-Espacenet (N = 0), Patentscope (N = 0), Play Store (N = 3), App Store (N = 0), and the Brazilian National Institute of Industrial Property (INPI) (N = 0). The following keywords were used in Portuguese, English, and Spanish: Projects, Psychosocial Rehabilitation and Mental Health and Apps/ Projects, Psychosocial Rehabilitation and Mental Health, and App/ Proyectos, Rehabilitación Psicosocial y Salud Mental y Aplicaciones. An advanced search was conducted by title and/or abstract and/or full text words, when available. As inclusion criteria, titles and/or abstracts of patents/apps that described a relationship with the development of “Psychosocial Rehabilitation Projects Apps” were included, and as exclusion criteria, patents/apps that did not meet the eligibility criteria and were written in languages other than Portuguese, English, and Spanish were excluded.

This research resulted in 1 patent and 6 apps, which, after applying the exclusion and eligibility criteria [66,67], resulted in 3 mental health apps. After installing the apps on personal smartphones (of two researchers) to learn about their features, the patent was read and excluded because it did not have an available app and there were no details available due to copyright restrictions. Two apps were also excluded due to interface defects that prevented the evaluation of their functionalities. Therefore, only 1 mental health app was included (Table 3), which was analyzed in terms of its functions and technological resources [47].

**Figure 2:**
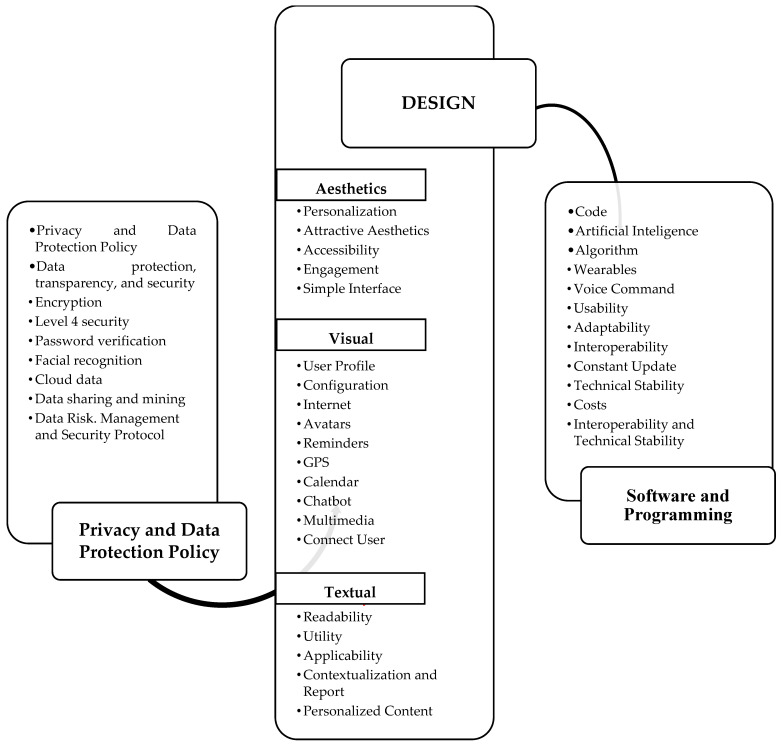
Presents the possible composition of the prototype “Psychosocial Rehabilitation Project App”. Source: Prepared by the authors, 2024 (see Figure S1).

Following Braun and Clarke’s (2006, 2022, 2023) six-phase approach to thematic analysis, we analyzed the articles and the app. Initially, we immersed ourselves in the data (phase 1), thoroughly reading and rereading the materials to gain a comprehensive understanding. This familiarization process led to the generation of initial codes (phase 2) related to functions, technological resources, and recommendations. These codes were then systematically grouped into potential themes and subthemes (phase 3), reflecting recurring patterns and significant meanings within the data. We then rigorously reviewed and refined these candidate themes (phase 4), ensuring they accurately represented the data and captured the nuances of the coded segments. This iterative process involved revisiting the data and codes to ensure coherence and comprehensiveness. Subsequently, we clearly defined and named the final themes and subthemes (phase 5), providing concise labels that encapsulated the essence of each. To enhance the trustworthiness and validity of our findings, a second researcher independently reviewed the identified themes and codes (phase 4), a process informed by best practices in collaborative thematic analysis. This independent verification helped to mitigate potential researcher bias and strengthened the rigor of our analysis [47]. Figure 2 presents the resulting themes, subthemes, and illustrative codes. This figure provides a clear and transparent overview of our analytical process and findings. It was created to visually encapsulate the structured approach and key themes identified, making the data more accessible and understandable. Guided by Braun and Clarke’s framework, this structured approach allowed us to systematically analyze the data and develop a rich understanding of the key themes present.

## 3. Results

Presented below are the themes and corresponding codes that refer to initial requirements to compose the prototyping of the “Psychosocial Rehabilitation Project App”. 

## 4. Discussion

For the prototyping of the “Psychosocial Rehabilitation Project App” it is imperative that researchers pay attention to the simplicity of its design [44,60], as a simple appearance and simple functionalities do not make it unviable and do not prevent its personalization. Personalization (background, themes, and colors) of an app allows the user to feel represented by and belonging to this app in a way that addresses diversities and gender representation and makes them feel included from its prototyping to its availability for download [40,42,49]. To make it attractive, there must be a configuration menu so that the user can edit profile information, choose avatars or photos, and make adjustments related to necessary functionality/update notifications [24,40,45,51]. Personalization should be planned and designed in advance in the app’s design.

The design needs to be user-centered and have content and technological resources that unfold into images, icons, and texts [46,56], buttons, and menus that meet their functionality and are based on theoretical support and/or scientific evidence [12,40,46], with the inclusion of colorful icons and appropriate screen sizes, a pleasant modern aesthetic, and needs to avoid cognitive overload [25,35,46,54,56].

Another important requirement is interactivity and ease of use [64], which favors effective participation and testing by the target audience [55]. Furthermore, it is essential that mental health apps have accessibility, which is expressed through the inclusion of diversities and human particularities, being a factor that influences engagement [41,43,49], which represents the user’s involvement in using a mental health app and performing their tasks/actions [58]. In this way, accessibility should be valued, since, in most cases, mental health apps have a barrier to their effectiveness in the real world due to a lack of involvement with the user because they are decontextualized from their reality [38].

However, research has shown that the prototype or development of a mental health app with all the technological resources currently available to researchers and developers tends to generate difficulties in its use among its users [24,38,60]. Among the technological resources indicated, the following stand out: internet access, which is the main source of research on health/mental health information for professionals and users [40,46,68]; mental health care reminders [24,49,52]; calendars with dates and management of schedules and appointments [55]; GPS to locate mental health services [12,35]; a chatbot, an automatic robot for interaction with users with pre-programmed prompts [61]; multimedia [24,53,56]; personalized feedback through prompts, which allow for rapid intervention; reports for the composition of metrics, and refined or raw data about their activities [42]; a resource for connecting with a therapist (through video calls, chat, and other media resources) [39,45]; gamification [10]; artificial intelligence [39,45,69] with the help of voice commands and multimedia (sound captures); and wearables that allow learning about human behavior [52] through the algorithm [39,45,48,50,69].

The stage at which researchers and developers choose the technological resources to be used in mental health apps is prototyping, and at this moment, the app’s design is improved and sketched, and subsequently, development proceeds, focusing on programming to activate the mental health app, enabling its interoperability, usability [53], and choice of operating system [56,70]. At this stage, it is also defined whether the code used will be open or not [36,53]. The programming of mental health apps requires special attention to adaptability and interoperability. Adaptability allows the app to be modified to meet the constantly evolving needs of users. Interoperability, in turn, ensures that the app can share data with other health systems, facilitating integration and access to relevant information. These characteristics are fundamental to ensuring the usability of the app and its ability to prevent technical problems, as pointed out by [25,58].

Regarding data security and privacy, the literature suggests a scarcity of scientific research on the full understanding of the risks and threats to data security and privacy in mental health apps. The main threats to privacy in these apps concern likability, identifiability, non-repudiation, detectability, information disclosure, unawareness, and non-compliance [71]. 

Studies [72,73,74,75] focus on privacy policies, others on a more bioethical approach [76,77] or specifically on data sharing [78], or recommendations for mental health app evaluation [79]. One study alerted us to the need for mental health apps to have clear policies on infrastructure, processing, storage, and the sharing of data [80].

Data privacy and security are also a barrier to the usability of mental health apps [50,81], requiring developers and researchers to implement security and risk management measures and strategies, and through interventions for cases of privacy invasion through data leakage [41,82]. Measures such as encryption [51], logout after five minutes of continuous non-use of the app by its user, password protection and password verification by email, smartphones, and/or facial or digital detection [38,45] can prevent privacy violations and data theft [6,35,44,55,56,76,77].

Often, the large amount of data produced by mental health apps is stored in the cloud [56] to reduce the memory load on the user’s smartphone and also ensure that data are not lost [40]. Generally, cloud services contracted from trusted companies are encrypted, but by outsourcing, the risk of data invasion increases, and control is lost by the app owners and the users themselves [76].

The study also warns that cloud infrastructure services, such as storage on Firebase, which guarantee the functionality of apps in an “easy” way for developers, limit the control over the data, and the process may not be transparent [80]. It is recommended that developers build a well-structured flow for the transmission, sharing, and storage of data, making them aware that mental health apps are potentially risky systems regarding data privacy and security, consulting lawyers when developing the security and privacy policy, knowing the policies and laws related to data privacy in their respective countries, collecting valid user consent, and receiving advice from professionals specialized in data security and privacy in relation to mental health apps [80].

Data mining has been used with the support of artificial intelligence to use data for mental health intervention, make diagnoses, and prevention, which is still a little-explored field and has been justified as a way to ensure quality in different aspects involving information, such as assessment, diagnoses, evolutions, records, and images produced in health [45,53].

The access to the internet through health applications faces the issue of data confidentiality [76,77,83]. This problem worsens when it comes to data produced in the field of mental health, which are sensitive and generally used as criteria for diagnosing mental disorders and their comorbidities [77]. In the prototype of the “Psychosocial Rehabilitation Project App”, researchers and developers intend to build a data protection and security policy with the assistance of a professional lawyer to address the ethical standards and conditions of use of the app. For this purpose, the authors’ recommendations will be used [77,79]. The concept of Netiquette—a virtual behavior protocol, similar to rules of conduct socially defined in the real world, with the objective of fostering a healthy, dynamic, and fluid coexistence on the internet, respecting people’s rights—will be encouraged among users by the data protection and security policy [84]. Users will be strongly discouraged from sharing patient data (without their authorization) with third parties, under penalty of copyright infringement, guaranteed by the app’s responsible parties, the exclusion of the profile, communication to professional councils, and police and judicial authorities for due action [6,76,77].

Thus, the prototype of the “Psychosocial Rehabilitation Project App”, as an idealized product, emerges anchored in the theory of psychosocial rehabilitation and the structure of the psychosocial rehabilitation project [1,2,3,85]. In developing this research it was found that there is no app in the consulted databases on mental health with the purpose of facilitating the management, construction, monitoring, and evaluation of psychosocial rehabilitation projects by mental health professionals.

In this sense, the prototyping of the “Psychosocial Rehabilitation Project App’” will be based on graphic design methodology, which allows researchers/developers to use subjective aspects, such as intuitiveness and professional experience, during the creative process of developing technological products [22,86,87]. The entire construction of the prototype will take place in the Marvel software [88], with the creative process guided by the pursuit of simplicity [60], translation, and dialog between technology and mental health [1]. This approach aims to balance its functionality with the design oriented by the artistic process of the researchers/developers [7,22]. 

Although this is both an opportunity for transformation and improvement in mental health care, it is also a great challenge, as there is an immense amount of technological resources that can be integrated into the “Psychosocial Rehabilitation Project App”, whether substantially in prototyping, and later, in development, requiring researchers and developers to make assertive choices that result in an easy-to-use app with moderate to high usability [24,38] that guarantees data security without violating human rights [38,55,76,89].

### Directions Futures

The next steps are aimed at developing the “Psychosocial Rehabilitation Project App”, based on the PCC (Population, Concept, and Context) framework [1]. The user population will consist of mental health professionals (e.g., psychiatric nurses, doctors, psychologists, therapists, etc.). The concept is the psychosocial rehabilitation project: a systematic care instrument based on the theory of psychosocial rehabilitation, defined with phases: assessment, goals, interventions, and agreements. The context will be the CAPS service (psychosocial rehabilitation care environment). To this end, a focus group will be conducted with the CAPS team to define the prototyping requirements contextualized to the work environment of these professionals, to be integrated into the design of the “Psychosocial Rehabilitation Project App”. Through the creative process and leap of the principal researcher, using the Marvel prototyping tool [22,88], this creative process will always be guided by the theoretical support of psychosocial rehabilitation and the structure defined in the literature of the psychosocial rehabilitation project [2,3,85], as can be seen in Table 4 below, which allows capturing the requirements defined in Figure 2, the findings of the present research which has been selected and adapted according to the process and creative leap, and in accordance with the structure of the psychosocial rehabilitation project [2,3,7,22,86,87]. In addition, the next research steps will use health professionals’ consultancy through prototype validation [16,23,24,90,91,92,93]. Finally, for the authors, the creative leap allows for changes, as the researchers’ creativity and other emerging senses can add changes and innovation to make the prototype of the “Psychosocial Rehabilitation Project App” coherent, adequate, and functional without compromising its future development and usability [22,24,56].

## 5. Conclusions

The prototyping of the “Psychosocial Rehabilitation Project App” should prioritize data security and protection, simplicity in design, and the integration of technological resources that facilitate the management, construction, monitoring, and evaluation of psychosocial rehabilitation projects by mental health professionals. The integrative review conducted in this work identified three main themes: privacy and data protection policy; design; and software and programming. These themes are crucial to ensure that the application meets the needs of mental health professionals and patients, making it important and justified to reflect on these themes before prototyping, which become initial prototyping requirements. The review provided a list of many technological resources and recommendations regarding data privacy, requiring researchers to reflect on their feasibility, so that their choices are aligned with the creative process and functionality of the “Psychosocial Rehabilitation Project App”, always guided by the psychosocial rehabilitation project structure.

Finally, a limitation of this study is the lack of practical validation of the identified requirements, which suggests the need for future studies to test and validate the effectiveness of the prototype in real-use contexts.

## Figures and Tables

**Table 1:** Description of the search expression, based on the indexed descriptors and their respective synonyms combined using the Boolean operators.

PICo	DeCs/MeSH	SYNONYMS
P	“Design de Software” OR “Software Design” OR “Diseño de Software”	“Desenho de Programas de Computador” OR “Diagrama de Bloco (Computação)” OR “Diagramas de Bloco (Computação)” OR “Fluxograma (Computação)” OR “Fluxogramas (Computação)” OR “Projeto de Programas de Computador” OR “Projeto de Software” OR “Design, Software” OR “Designs, Software” OR “Software Designs” OR “Flowcharts (Computer)” OR “Flowchart (Computer)” OR “Flow Charts (Computer)” OR “Chart, Flow (Computer)” OR “Charts, Flow (Computer)” OR “Flow Chart (Computer)” OR “Flowcharts” OR “Flowchart” OR “diagrama de flujo (informática)” OR “diagramas de flujo (informática)” OR “diseño de programas Informáticos”
	OR	
	“Projeto de Sistemas” OR “System Design” OR “Diseño de Sistemas”	Software OR “Análise de Sistemas” OR “Digital Technologies” OR “Technologies, Digital” OR “Technology, Digital” OR “Digital Electronics” OR “Electronics, digital”
	OR	
	“Software” OR “Software” OR “Programas Informáticos”	“Engenharia de Software” OR “Ferramentas de Software” OR “Programas de Computador e Programação” OR “Programas de Computação” OR “Programas para Computadores” OR “Programação e Programas de Computador” OR “Software Proprietário” OR “Software de Aplicativos” OR “Software de Aplicativos de Computador” OR “Software de Aplicações” OR “Software de Aplicações Informáticas” OR “Suporte Lógico de um Sistema Informático” OR “Computer Software” OR “Software, Computer” OR “Open Source Software” OR “Open Source Softwares” OR “Software, Open Source” OR “Softwares, Open Source” OR “Source Software, Open” OR “Source Softwares, Open” OR “Computer Programs” OR “Computer Program” OR “Program, Computer” OR “Programs, Computer” OR “Software Tools” OR “Software Tool” OR “Tool, Software” OR “Tools, Software” OR “Software Engineering” OR “Engineering, Software” OR “Computer Applications Software” OR “Applications Software, Computer” OR “Applications Softwares, Computer” OR “Computer Applications Softwares” OR “Software, Computer Applications” OR “Softwares, Computer Applications” OR “Computer Software Applications” OR “Application, Computer Software” OR “Applications, Computer Software” OR “Computer Software Application” OR “Software Application, Computer” OR “Software Applications, Computer” OR “Computer Programs and Programming” OR “aplicaciones de software” OR “aplicaciones de software informático” OR “aplicación de software” OR “herramientas de software” OR “ingeniería de software” OR “programas de computadora y programación” OR “programas de ordenador” OR “programas Informáticos” OR “programas informáticos y programación” OR “programas para computadoras” OR “programas para ordenadores” OR “software de aplicaciones informáticas” OR “software de código abierto” OR “software informático” OR “soporte lógico de un sistema informático”
AND		
I	“Aplicativos Móveis” OR “Mobile Applications” OR “Aplicaciones Móviles”	“Aplicativos Eletrônicos Portáteis” OR “Aplicativos de Software Portáteis” OR “Aplicativos em Dispositivos Móveis” OR “Aplicativos para Dispositivos Móveis” OR “Apps Móveis” OR “Application, Mobile” OR “Applications, Mobile” OR “Mobile Application” OR “Mobile Apps” OR “App, Mobile” OR “Apps, Mobile” OR “Mobile App” OR “Portable Software Apps” OR “App, Portable Software” OR “Portable Software App” OR “Software App, Portable” OR “Portable Software Applications” OR “Application, Portable Software” OR “Portable Software Application” OR “Software Application, Portable” OR “Smartphone Apps” OR “App, Smartphone” OR “Apps, Smartphone” OR “Smartphone App” OR “Portable Electronic Apps” OR “App, Portable Electronic” OR “Electronic App, Portable” OR “Portable Electronic App” OR “Portable Electronic Applications” OR “Application, Portable Electronic” OR “Electronic Application, Portable” OR “Portable Electronic Application” OR “aplicaciones de software portátiles” OR “aplicaciones electrónicas portátiles” OR “aplicaciones Móviles” OR “aplicaciones para móviles” OR “aplicaciones para smartphones” OR “aplicaciones para teléfonos inteligentes” OR “aplicaciones portátiles de software” OR “aplicación móvil” OR “softwares portátiles”
	AND	
	“Projetos” OR “Projects” OR “Proyectos”	“Projeto” OR “Projects” OR “Proyectos”
	AND	
	“Reabilitação Psiquiátrica” OR “Psychiatric Rehabilitation” OR “Rehabilitación Psiquiátrica”	“Atenção Psicossocial” OR “Rehabilitation, Psychiatric” OR “Mental Health Rehabilitation” OR “Health Rehabilitation, Mental” OR “Rehabilitation, Mental Health” OR “Psychosocial Rehabilitation” OR “Rehabilitation, Psychosocial” OR “Psychosocial Care” OR “Care, Psychosocial” OR “Cares, Psychosocial” OR “Psychosocial Cares” OR “atención Psicosocial”
AND		
Co	“Saúde Mental” OR “Mental Health” OR “Salud Mental”	“Higiene Mental” OR “Área de Saúde Mental” OR “Health, Mental” OR “Mental Hygiene” OR “Hygiene, Mental” OR “higiene mental”

Source: Prepared by the author (2024).

**Table 2:** Selected articles for the integrative review.

Articles	Resume	Main Finding (Requirements for Prototyping)
González-Pérez, A., Matey-Sanz, M., Granell, C., Díaz-Sanahuja, L., Bretón-López, J., and Casteleyn, S. (2023). AwarNS: A framework for developing context-aware reactive mobile applications for health and mental health. *Journal of Biomedical Informatics*, 141(104359), 1–20. https://doi.org/10.1016/j.jbi.2023.104359 [36].	Theoretical study that narrates an open-source platform (AwarNS) to develop an app (its structure) in mental health to prevent technical failures and ensure data privacy by developers.	- Privacy Policy.
Gaebel, W., Lukies, R., Kerst, A., Stricker, J., Zielasek, J., Diekmann, S., Trost, N., Gouzoulis-Mayfrank, E., Bonroy, B., Cullen, K., Desie K., Ewalds Mulliez, A. P., Gerlinger, G., Günther, K., Hiemstra, H. J., McDaid, S., Murphy, C., Sander, J., Sebbane, D., Roelandt, J. L., Thorpe, L., … Vlijter, O. (2021). Upscaling e-mental health in Europe: a six-country qualitative analysis and policy recommendations from the eMEN project. *European Archives of Psychiatry and Clinical Neuroscience*, 271(6), 1005–1016 [37].	A narrative literature review and survey study that aimed to identify the barriers to the implementation of mHealth in Europe. These are related to concerns regarding the privacy and protection of psychiatric patient data.	- Privacy and Data Protection Policy.
Wu, A., Scult, M. A., Barnes, E. D., Betancourt, J. A., and Falk, A. (2021). Gunning FM. Smartphone apps for depression and anxiety: a systematic review and meta-analysis of techniques to increase engagement. *NPJ Digital Medicine*, 4(1), 1–9 [38].	A systematic literature review/meta-analysis study (N = 25 articles) suggests that a mental health app needs to have aspects related to content (texts and images), security and privacy policies (security and privacy policy, encryption, and password verification), and accessibility (free or not) in its design.	- Content (texts/or images) related to the purpose of the mental health app. - Security and privacy policies. - Encryption and password verification. - Accessibility (free or not).
Hariman, K., Ventriglio, A., and Bhugra, D. (2019). *The Future of Digital Psychiatry. Current Pyschiatry Reports*, 21(9), 88 [39].	Theoretical study analyzes the influence of digital technologies (telepsychiatry, videoconferencing, apps, artificial intelligence, and social media) on psychiatry.	- Icon for videoconferencing to discuss mental health cases by the team. - Icon for telepsychiatry for consultation of professionals and patients. - Simple interface that allows a pleasant experience and user engagement.
Ramos, G., Ponting, C., Labao, J. P., and Sobowale K. (2021). Considerations of diversity, equity, and inclusion in mental health apps: A scoping review of evaluation frameworks. *Behaviour Research and Therapy*, 147, 103990 [40].	A scope review study with a sample of 43 articles aimed to assess whether the structures of the applications meet the criteria of inclusion, equity, and diversity.	- Have an attractive design/aesthetics, functionality and clinical validity, interactive content, engagement, ease of learning, comprehensibility/simple language, and meet ethical standards (privacy, security and data transparency), usability, applicability, contextualization with the user’s real world, possibility of user customization and feedback. - Access (internet connectivity, data usage, data input and output, maintenance cost and Android operating system). - Content: accessible language, literacy, content adapted to the reality and context of users, personalization/avatars, adapted to people with disabilities. - Appearance: visibility and legibility of texts, icons, buttons, menus and screen content, diverse design that represents the user, including aspects of gender and sexuality. The appearance should give users the feeling that they are welcome.
Huckvale, K., Nicholas, J., Torous, J., and Larsen, M. E. (2020). Smartphone apps for the treatment of mental health conditions: status and considerations. *Current Opinion in Psychology*, 36, 65–70 [41].	Theoretical study that addresses aspects related to mental health apps, such as their basis in scientific evidence, privacy and data security, engagement, and that in the construction of a mental health app, the human component and its applicability to the real world (contextualization) are considered.	- Data privacy policy (transparency with confidentiality, data processing, and data security). - Data encryption. - Data risk management and security protocol. - The quality of the PRP app is defined by the evidence, security, data protection, usability, accessibility, interoperability, and technical stability. - The content of PRP apps should be evidence-based. - User engagement happens if the PRP app is contextualized and applied to their clinical reality.
Hanssen, E., Balvert, S., Oorschot, M., Borkelmans, K., van Os, J., Delespaul, P., and Fett, A. K. (2020). An ecological momentary intervention incorporating personalised feedback to improve symptoms and social functioning in schizophrenia spectrum disorders. *Psychiatry Research*, 284, 112695 [42].	Intervention study (N = 50 schizophrenic patients) that evaluated the effectiveness of the SMART app regarding its ability to offer personalized feedback to schizophrenic patients, demonstrating to be effective in reducing psychotic symptoms and loneliness.	- The PRP app needs to be useful, easy to use, attractive, and personalized to the user. - Personalized feedback to psychiatric patients to deal with the problems and limitations resulting from the mental disorder. - Data storage in the cloud.
Li, H., Lewis, C., Chi, H., Singleton, G., and Williams, N. (2020). Mobile health applications for mental illnesses: An Asian context. *Asian Journal of Psychiatry*, 54(102209), 1–6 [12].	A narrative literature review study (N = 22) conducted to track publications that identified mental health applications developed and/or used in an Asian context.	- The PRP app should be contextualized and adapted to the user’s culture. Regarding the structure, icons/buttons for videoconferencing for referral/consultation of the psychiatric patient, and GPS to locate services to refer during the treatment plan.
Lemon, C., Huckvale, K., Carswell, K., and Torous, J. (2020). A narrative review of methods for applying user experience in the design and assessment of mental health smartphone interventions. *International Journal of Technology Assessment in Health Care*, 36(1), 64–70 [43].	A narrative literature review study that addresses the importance of user experience both in the development of the application and during its use, in order to improve it.	- The development of a PRP app should consider the user experience in its prototyping and use (favoring a participatory interaction that allows gathering feedback and insights). - The PRP app should be evaluated for its satisfaction, acceptability, feasibility, usefulness, friendliness, learnability, credibility and usability. - The PRP app should collect continuous feedback from its users when available. Having a user evaluation tool, whether qualitative and/or quantitative (metrics and comments).
Torous, J., and Vaidyam, A. (2020). Multiple uses of app instead of using multiple apps- a case for rethinking the digital health technology toolbox. *Epidemiology and Psychiatric Sciences*, 29, e100 [44].	Theoretical study that describes the MindLAMP platform (which provides mental health applications to users after a prior assessment).	- Consider the global impact, whether it is open source, simplicity, efficiency, ethics, resource, and data privacy. - Assess and understand the risks. - Personalizing the app means meeting with mental health professionals or/users. - Create exclusive and data-driven interventions.
Miller, E., and Polson, D. (2019). Apps, Avatars, and Robots: The Future of Mental Healthcare. *Issues in Mental Health Nursing*, 40(3), 208–214 [45].	Theoretical study that critically reflects on how digital technologies can impact positively (access and social inclusion) and/or negatively (feelings, expressions, and reception are not human) in mental health care.	- As a resource to be used in an application: artificial intelligence (data mining and support for interventions to mental health professionals), patient self-monitoring, voice commands, personalization by avatars, and facial detection (security feature).
Bucci, S., Schwannauer, M., and Berry, N. (2019). The digital revolution and its impact on mental health care. *Psychology and Psychotherapy*, 92(2), 277–297 [46].	Theoretical study that addresses the impacts of digital tools on health. Considering positive points for being low cost, breaking geographical barriers to access to the health service, and negative points for the absence of theory that explains how the application works (psychological theories) and bioethical recommendations.	- Be based on theory and have its operation explained by this theory. - Reduce cognitive overload (content/information). - Safe handling and storage of data.
Ng, M. M., Firth, J., Minen, M., and Torous, J. (2019). User engagement in mental health apps: A review of measurement, reporting, and validity. *Psychiatric Services* (Washington, D.C.), 70(7), 538–544 [47].	Systematic literature review study (N = 40) that verifies the engagement potential of mental health apps. It considers that this metric is composed of usability, user satisfaction, acceptability and feasibility.	- Attractive and engagement the user (engagement). - Intersection with technology, mental health and user experience.
Kenny, R., Fitzgerald, A., Segurado, R., and Dooley, B. (2020). Is there an app for that? A cluster randomised controlled trial of a mobile app–based mental health intervention. *Health Informatics Journal*, 26(3), 1538–1559 [48].	Intervention study with 560 adolescents divided into two groups to evaluate the effectiveness of the CopeSmart app (coping strategies), which was not considered effective, in four weeks of use, to provide psychological well-being.	- Locate mental health support services. - Patient self-monitoring. - Personalized feedback from mental health professionals.
Quintana, M., Anderberg, P., Sanmartin Berglund, J., Frögren, J., Cano, N., Cellek, S., Zhang, J., and Garolera, M. (2020). Feasibility-usability study of a tablet app adapted specifically for persons with cognitive impairment—smart4md (Support monitoring and reminder technology for mild dementia). *International Journal of Environmental Research and Public Health*, 17(18), 6816 [49].	A pilot study that tests the feasibility and usability of the SMART4MD app (care management for elderly people with dementia), demonstrating that before making an app available, it is important to test it as much as possible to avoid “bugs”.	- User satisfaction interviews. - Reminders about health care (appointments, medication, and intervention). - Sharing of information.
Nordgreen, T., Rabbi, F., Tørresen, J., Skar, Y. S., Guribye, F., Inal, Y., Flobakk, E., Wake, J. D., Mukhiya, S. K., Aminifar, A., Myklebost, S., Lundervold, A. J., Kenter, R., Hammar, Å., Nordby, E., Kahlon, S., Tveit Sekse, R. J., Griffin, K. F., Jakobsen, P.,… and Lamo, Y. (2021). Challenges and possible solutions in cross-disciplinary and cross-sectorial research teams within the domain of e-mental health. *Journal of Enabling Technologies* (JET), 15(4), 241–251 [50].	Experience report study addressing the main challenges and solutions for prototyping a mental health app. It considers important for the development of an app in this area: teamwork, participatory person-centered design, efficient communication between developer and mental health professional/researcher, and desire/willingness to learn from each other.	- Simple design. - Clinical content, software, hardware, and data sets. - Participation of the mental health team in the prototyping of the PRP app. - Interoperability. - Data Security and Privacy.
Mukhiya, S. K., Lamo, Y., and Rabbi, F. (2022). A Reference Architecture for Data-Driven and Adaptive Internet-Delivered Psychological Treatment Systems: Software Architecture Development and Validation Study. *JMIR Human Factors*, 9(2), e31029 [51].	Experience report study describing the prototype, development, and evaluation of a reference architecture (RA) for mental health app software. The RA should have adaptability, scalability, security, and modifiability.	- The operating system/architecture of the PRP app needs to have: - Interoperability. - Adaptability (possibility to make continuous improvements, not to be inflexible). - Creation of profile/user. - Level 4 security: BankID, Microsoft Azure, or Amazon Web Services. - Contents, presentation/design, feedbacks, reminders, and usability.
Alqahtani, F., Al Khalifah, G., Oyebode, O., and Orji, R. (2019). Apps for Mental Health: An Evaluation of Behavior Change Strategies and Recommendations for Future Development. *Frontiers in Artificial Intelligence*, 2(30), 1–11 [52].	Mixed-methods study that presents recommendations on tools with persuasive strategies to be used in the development of a mental health app to improve its adherence and usability by future users.	- Credibility (provide scientifically proven and evidence-based information). - Social support. - Support for tasks. - Self-monitoring. - Performance feedback/rewards (coins and trophies). - Personalization (background, images, themes, sounds according to user’s preferences). - Reminders to professionals about actions or interventions related to the patient’s PRP. - Data Security and Privacy.
Timakum, T., Xie, Q., and Song, M. (2022). Analysis of E-mental health research: mapping the relationship between information technology and mental healthcare. *BMC Psychiatry*, 22(1), 57 [53].	Bibliometric study (N = 3172 articles) with the purpose of mapping the beginning of publications and trends of digital technologies in mental health. Furthermore, it emphasizes the importance of interdisciplinary work in the development of mental health apps.	- Programming (machine learning, algorithms, data mining, Android, and artificial intelligence). - Data Privacy and Security. - Contents (multimedia (videos, email, social media, text messages, and chats). - Design with interactive interface. - Usability. - Integrative Practices.
Bianco, C. L., Myers, A. L., Smagula, S., and Fortuna, K. L. (2021). Can Smartphone Apps Assist People with Serious Mental Illness in Taking Medications as Prescribed? *Sleep Medicine Clinics*, 16(1),213–222 [54].	Theoretical study that presents mental health apps that are used to improve medication adherence in psychiatric patients.	- User-centered design that considers the needs and suggestions of users. This will provide greater engagement and acceptability of the PRP app by mental health professionals. - Interventions: psychoeducation, mood monitoring, sleep and social functioning, and medication adherence.
Bush, N. E., Armstrong, C. M., and Hoyt, T. V. (2019). Smartphone apps for psychological health: A brief state of the science review. *Psychological Services*, 16(2), 188–195 [55].	Theoretical study that critically analyzes apps in psychology, which should be verified for the quality of their content and have scientific evidence that proves their efficacy and clinical effectiveness for use.	- Content quality (scientific evidence). - Prototyping, consulting and participation of end-users. - Data privacy and security (end-to-end encryption) and ethical considerations. - Functionality: Diagnosis, Treatment, Monitoring, Prescription/Intervention, and Evaluation. - Appointment reminders/calendar management. - Telehealth. - Algorithm. - Artificial intelligence. - Wearables. - Cognitive-Behavioral Therapy.
Callan, J. A., Jacob, J. D., Siegle, G. J., Dey, A., Thase, M. E., Dabbs, A. D., Kazantzis, N., Rotondi, A., Tamres, L., Van Slyke, A., and Sereika, S. (2021). CBT MobileWork©: User-Centered Development and Testing of a Mobile Mental Health Application for Depression. *Cognitive Therapy and Research*, 45(2), 287–302 [56].	Study describes the 2-phase development and validation of the app (development: 8 depressed patients and 5 therapists, and testing: 5 therapists and 15 depressed patients) CBT MobileWork© to streamline the homework tasks proposed by cognitive-behavioral therapists in depressed patients.	- User-centered design (iterative methodology that seeks the active participation of users). - Design features: Adequate screen size and audiovisual capacity (colorful icons/YouTube™). - Android™ platform (fast and easy development). - Content: aligned with theory, clinical recommendations and evidence-based intervention. - Integrate users’ daily activities. - User feedback. - Data encryption (logout after five minutes of non-use protects against indiscriminate privacy invasion and data theft).
Chan, S., Li, L., Torous, J., Gratzer, D., and Yellowlees, P. M. (2019). Review and Implementation of Self-Help and Automated Tools in Mental Health Care. *The Psychiatric clinics of North America*, 42(4), 597–609 [57].	Theoretical study that critically analyzes information technologies used in mental health, demonstrating their potential, as well as the shortcomings that need to be overcome.	- Data privacy and security (encryption). - User-centered design. - Clinical content. - Technological resources: artificial intelligence, feedbacks, communication recognition of speech, facial expression, voice and text, emails, voicemails, telepsychiatry, video, podcast, YouTuber, chatbot, voice assistant, and GPS.
Chan, A. H. Y., and Honey, M. L. L. (2022). User perceptions of mobile digital apps for mental health: Acceptability and usability-An integrative review. *Journal of Psychiatric and Mental Health Nursing*, 29(1), 147–168 [35].	Integrative literature review study (N = 17 articles) that identified users’ perceptions of mental health apps (mental health team knows the patient better, suggestions for app improvements, satisfaction with the app, and technical problems with app use.	- Data Security and Privacy.
Birrell, L., Furneaux-Bate, A., Debenham, J., Spallek, S., Newton, N., and Chapman, C. (2022). Development of a Peer Support Mobile App and Web-Based Lesson for Adolescent Mental Health (Mind Your Mate): User-Centered Design Approach. *JMIR Formative Research*, 6(5), e36068 [24].	Study describes the development of the Mind Your Mate app that psychoeducates adolescents about anxiety, depression, and psychoactive substance use.	- Intervention: social support. - Privacy Policy, Transparency and Data Security. - Customizable Resources. - Configuration (user can edit profile and notification information). - Reminders. - Technological failures: Ads, delays, batteries, and difficulties in using.
Rosenfeld, E. A., Lyman, C., and Roberts, J. E. (2022). Development of an mHealth App–Based Intervention for Depressive Rumination (RuminAid): Mixed Methods Focus Group Evaluation. *JMIR Formative Research*, 6(12), e40045 [25].	Mixed-methods study that describes the prototyping and validation of the RuminAid app prototype to reduce rumination in depressed patients.	- Design with modern aesthetics and attractive color scheme. - Gamification.
Lipschitz, J. M., Van Boxtel, R., Torous, J., Firth, J., Lebovitz, J. G., Burdick, K. E., and Hogan, T. P. (2022). Digital Mental Health Interventions for Depression: Scoping Review of User Engagement. *Journal of Medical Internet Research*, 24(10), e39204 [58].	Scoping review study (N = 22 articles) aimed at defining metrics to measure user engagement with mental health apps.	- The engagement report demonstrates user involvement with the mental health app and is composed of: usage metric (quantitative of the downloaded app divided by the effective usage time of the app) and adherence metric (number of app tasks divided by the total number of tasks to be completed).
Burchert, S., Alkneme, M. S., Bird, M., Carswell, K., Cuijpers, P., Hansen, P., Heim, E., Harper Shehadeh, M., Sijbrandij, M., Van’t Hof, E., and Knaevelsrud, C. (2019). User-centered app adaptation of a low-intensity e-mental health intervention for Syrian refugees. *Frontiers in Psychiatry*, 9, 663 [59].	Multi-method study (N = 128 participants) conducted in 3 phases with the aim of developing the interactive prototype of the Step-by-Step app for mental health intervention focused on the Syrian ethnic refugee population in Europe and the Middle East.	- Usability. - Use interactive prototyping software, suggests InVision (InVisionApp inC).
Hoffman, L., Benedetto, E., Huang, H., Grossman, E., Kaluma, D., Mann, Z., and Torous, J. (2019). Augmenting mental health in primary care: A 1-Year Study of Deploying Smartphone Apps in a Multi-site Primary Care/Behavioral Health Integration Program. *Frontiers in Psychiatry*, 10, 94 [60].	Intervention study that developed and tested (N = 32 mental health professionals) the mental health app toolkit to support the health team to recommend them to patients.	- App needs to be constantly updated. - Simplicity in Design. - Apps with limited numbers of concepts, functions/activities may be more attractive than those with multiple features.
Kruzan, K. P., Reddy, M., Washburn, J. J., and Mohr, D. C. (2022). Developing a Mobile App for Young Adults with Nonsuicidal Self-Injury: A Prototype Feedback Study. *International Journal of Environmental Research and Public Health*, 19(23), 16163 [61].	Qualitative approach study (N = 10 participants) aimed at prototyping an app for self-harm prevention in adolescents and young adults.	- Simple structure. - Easy to use. - Chatbot. - Avatars.
Borghouts, J., Eikey, E., Mark, G., De Leon, C., Schueller, S. M., Schneider, M., Stadnick, N., Zheng, K., Mukamel, D., and Sorkin, D. H. (2021). Barriers to and facilitators of user engagement with digital mental health interventions: systematic review. *Journal of Medical Internet Research*, 23(3), e24387 [62].	Literature review study (N = 208 articles) that seeks to identify the barriers and facilitators for mental health apps to have engagement among their users.	- Engagement is facilitated by the credibility of the mental health app. - Demographic variables for users (name, age, gender, education, profession, etc.). - Contextualization with the user’s professional reality. - Content consistent with the proposed intervention. - Privacy and confidentiality. - Usability. - Costs of developing the mental health app.
Chow, P. I. (2020). Developing Mental or Behavioral Health Mobile Apps for Pilot Studies by Leveraging Survey Platforms: A Do-it-Yourself Process. *JMIR mHealth and uHealth*, 8(4), e15561 [18].	Guides researchers on how to develop a mental health app according to 5 development stages (theoretical structuring, systematization of content and appearance, posting on Google Play Store, and pilot testing), which he considered very laborious and stressful.	- Theoretical structure that underlies the app’s intervention. - Content and appearance.
Marciniak, M. A.; Shanahan, L.; Rohde, J.; Schulz, A.; Wackerhagen, C.; Kobylińska, D. et al. Standalone Smartphone Cognitive Behavioral Therapy–Based Ecological Momentary Interventions to Increase Mental Health: Narrative Review. *JMIR mHealth and uHealth*, v. 8, n. 11, p. 1–46, 12 nov. 2020 [63].	Literature review study (N = 20 studies) that investigates mental health apps developed with concepts from Cognitive-Behavioral Therapy.	- Content. - Feasibility. - Efficacy and effectiveness.
McCall, T., Ali, M. O., Yu, F., Fontelo, P., and Khairat, S. (2021). Development of a Mobile App to Support Self-management of Anxiety and Depression in African American Women: Usability Study. *JMIR Formative Research*, 5(8), e24393 [64].	Study evaluates the usability (N = 15 participants) of an app prototype to treat depression and anxiety in black women.	- Ease of use and interactivity. - Connect user to therapist. - Avoid bugs and poor interface design.
Martinengo, L., Stona, A. C., Griva, K., Dazzan, P., Pariante, C. M., von Wangenheim, F., and Car, J. (2021). Self-guided Cognitive Behavioral Therapy Apps for Depression: Systematic Assessment of Features, Functionality, and Congruence with Evidence. *Journal of Medical Internet Research*. 23(7), e27619 [65].	Literature review study (search in virtual stores for mental health apps) that evaluates the evidence and safety of available apps for depression treatment.	- Content. - Credibility. - Ease of use. - Data privacy and authentication.
Lopes, R. P., Barroso, B., and Deusdado, L. (2021). Digital technologies for innovative mental health rehabilitation. *Electronics* (Switzerland), *10*(18), 2260 [10].	Study that prototyped and developed a mental health app for the psychosocial rehabilitation of patients with schizophrenia. The design (dashboard) and technological resources were guided by gamification with virtual reality, in order to systematize the rehabilitation sessions.	- Gamification.

Source: Prepared by the author (2024).

**Table 3:** Selected applications for the search, app, and description platform.

Platform	App	Main Finding (Requirements for Prototyping)
Play Store	ACAPEF (app)	1. Home: Córdoba Association to help psychiatric patients with diagnosis and their family (icon of parents and two children); 2. news: asks if the user wants to receive news and notifications; 3. guide for the family; 4. categories; 5. bibliography; 6. ACAPEF audios; 7. recommended films; YouTube videos; and 8. share app.

Source: Prepared by the author (2024).

**Table 4:** Initial requirements for the prototyping of the psychosocial rehabilitation project app.

**Design**	
Aesthetics	Design with a color palette ranging from beige to gray, providing visual contrast for the user. Intuitive features, or with a clear description of their functionality (what it does). Users can customize the app with their photo/avatar. The design adapts to all operating systems and user hardware without losing its features and visualization.
Visual	- Home (multimedia video explaining the psychosocial rehabilitation project). - Projects (create and view the created projects). - Progress (insert or view progress). - Resources (edit profile, help and support, and technical information).
Textual	- Home (opening phrase: “Permeating life projects with meanings and significances built in the habitat, social network, and work”, and tips on psychosocial rehabilitation). - Projects (based on the structure of the psychosocial rehabilitation project: evaluation, diagnoses, goals, agreements, and interventions). - Progress (text editing). - Resources (credible content, contextualized to the Brazilian public health system).
**Software and Programming**	
Background technology	Webapps are types of applications recommended for their ability to allow compatibility with any operating system, to be used through a link by the user [82].
Functions	- Share (generate link for a PDF, print or save on hardware/cloud, or share via WhatsApp, email, etc.). - User support (consult references on psychosocial rehabilitation, mental health legislation, productivity records, usage metrics, and contact with developers/researchers). - Mental health support services (research on services in the Brazilian psychosocial network). - Add a new psychosocial rehabilitation project (possibility to add a new project using patient care data). - Search patient/project (search the patient’s project by name, facilitating its location among several constructed projects).
**Privacy and Data Protection Policy**	
Security and privacy policy (legal term regarding the use of the app and responsibilities). data flow diagram (DFD) in compliance with the Brazilian data protection law (Law nº 13.709/2018). Follow the bioethical recommendations of the study [77,79]. Encryption of stored data at level 4 [51], so that not even researchers and developers can see the data entered by third parties. Password recovery and verification via email and mobile phone. Perform security analysis of the app with tools such as MobSF v4.3.1 and Android Genymotion 4 (https://www.genymotion.com/, accessed on 9 February 2025), WebFX (https://www.webfx.com/web-development/services/website-security-analysis/, accessed on 9 February 2025), CLAUDETTE (http://claudette.eui.eu/ToS.zip, accessed on 9 February 2025), and PrivacyCheck (https://chromewebstore.google.com/detail/privacycheck/poobeppenopkcbjejfjenbiepifcbclg?pli=1, accessed on 9 February 2025) [80].	

Source: Prepared by the author (2024).

## Data Availability

Material Synthesis Literature Review can be found at Resultado RIL.pptx.

## Supplementary Materials

The following supporting information can be downloaded at: https://www.mdpi.com/article/10.3390/ijerph22020310/s1, Figure S1: “APP PROJETO DE REABILITAÇÃO PSICOSSOCIAL” (2024).
