# Supplementary material for: Initial Requirements for the Prototyping of an App for a Psychosocial Rehabilitation Project: An Integrative Review
Source: Int J Environ Res Public Health. 2025 Feb 18;22(2):310. doi: 10.3390/ijerph22020310 (PMC11855392; doi:10.3390/ijerph22020310)
Supplement: Supplementary file 1 [file ijerph-22-00310-s001.zip › ijerph-3377847-supplementary.pdf]

Supplementary Materials

1. Data Description

Figure 1 presents complete data from the literature review to compose Figure 2 presented in the article: Initial Requirements for the Prototyping of an App for a Psychosocial Rehabilitation Project: An Integrative Review.

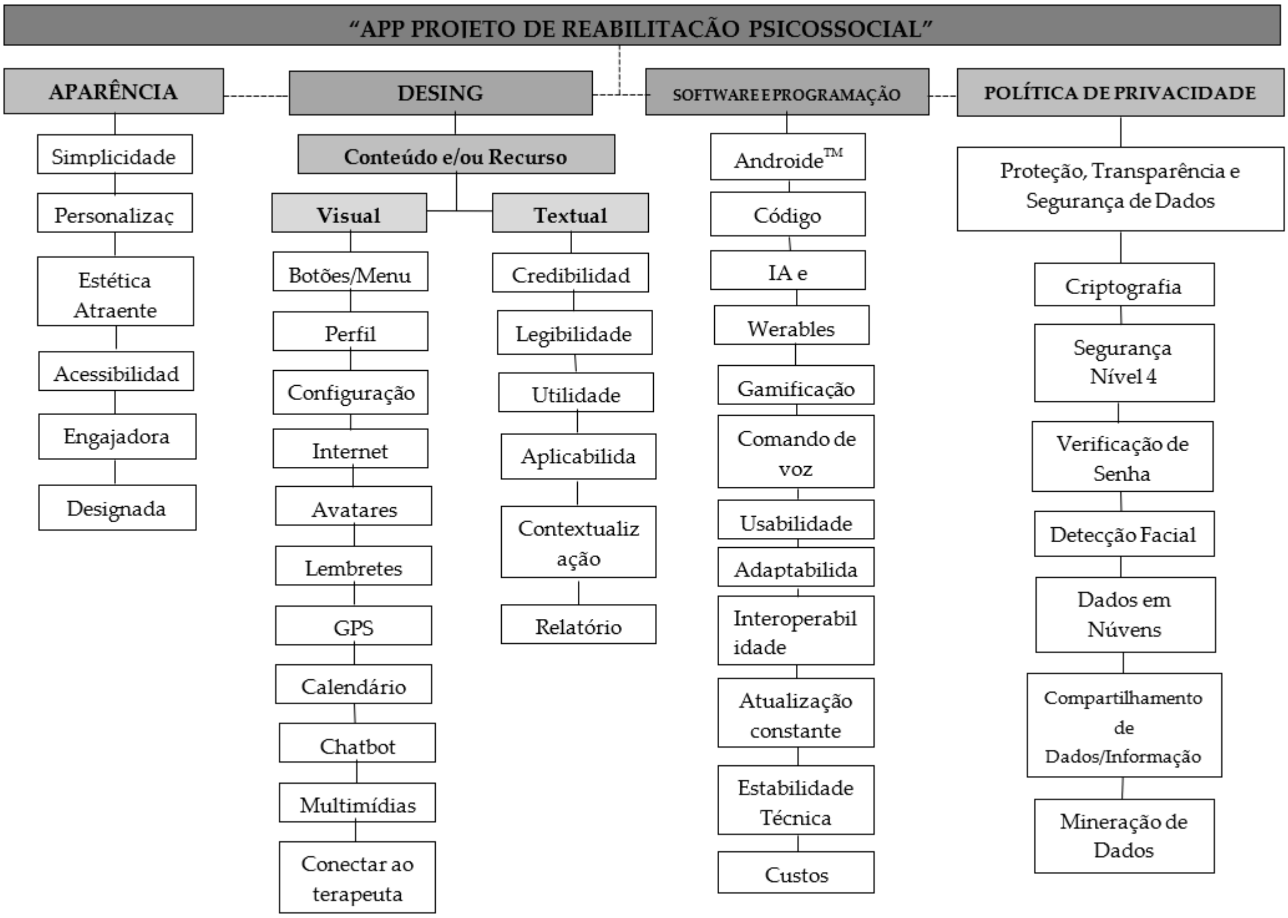

Figure S1. "APP PROJETO DE REABILITAÇÃO PSICOSSOCIAL" (2024).
